# Motivation factors affecting the job attitude of medical doctors and the organizational performance of public hospitals in Warsaw, Poland

**DOI:** 10.1186/s12913-020-05573-z

**Published:** 2020-07-29

**Authors:** Malgorzata Chmielewska, Jakub Stokwiszewski, Justyna Filip, Tomasz Hermanowski

**Affiliations:** 1 Department of Applied Toxicology, Division of Forensic Pharmacy, Pharmacy Division, Medical University of Warsaw, 81, Żwirki i Wigury Str, 02-091 Warsaw, Poland; 2 National Institute of Public Health – National Institute of Hygiene, 24, Chocimska Str, 00-791 Warsaw, Poland

**Keywords:** Performance feedback, Supervision, Management, Motivation, Attitude to work, Organizational performance

## Abstract

**Background:**

This paper examines the relationship between selected motivation factors that affect the attitude to work among medical doctors at public hospitals and the organizational performance of hospitals.

**Methods:**

This study was based on World Health Organization questionnaires designed to estimate motivation factors according to Herzberg’s motivation theory and to measure the level of organizational performance of hospitals by using the McKinsey model. A survey was conducted among physicians (*n* = 249) with either surgical (operative) or nonsurgical (conservative) specialty in 22 departments/units of general public hospitals in Warsaw, Poland.

The relationship between the chosen job motivation factors and organizational effectiveness was determined using Spearman’s rank correlation. Furthermore, 95% confidence intervals were calculated. The independent samples t-test was used to confirm statistically significant differences between the independent groups. Normality of the data was tested by the Kolmogorov–Smirnov test.

**Results:**

The survey revealed that motivation factors related to “quality and style of supervision” have the highest effect on the organizational performance of hospitals (Spearman’s rank correlation coefficient = 0.490; *p* < 0.001), whereas “performance feedback” has the lowest effect on organizational performance according to the surveyed healthcare professionals (54% of physicians).

**Conclusion:**

The principles of Individual Performance Review should be incorporated into strategies designed to improve the organizational performance of hospitals (with NHS serving as a potential role model) in order to establish specific rules on how to share performance feedback with individual physicians. The present study contributes to literature on human resource management in the healthcare sector and highlights the importance of nonfinancial aspects in improving the organizational performance of hospitals.

## Background

Work motivation and the relationship between job motivation and individual performance are one of the key issues investigated in studies on organizational behavior and human resources (HR) management [1].

HR are the main cost item in healthcare systems worldwide. Furthermore, the major and still growing proportion of healthcare funds is absorbed by in-patient hospital care [2]. According to the World Health Organization (WHO), almost 70% of overall healthcare costs can be attributed to in-patient services [3]. The available data indicate that the best approach to rationalize the costs of in-patient care is to improve the use of the available HR [4].

Many countries implement solutions that are not based on scientific evidence or empirical research. This trial-and-error approach, however, does not make it easy to choose rationally or take appropriate decisions [5].

Hospital performance largely depends on the way a hospital is managed and how it operates as a whole [6]. In many cases, literature review showed that hospital performance is largely determined by the engagement of medical staff, especially in terms of enhancing the organizational effectiveness of a hospital [7–10].

In the light of the above findings, it appears desirable to investigate the level of motivation among medical doctors working in public hospitals, their expectations in professional life, their job satisfaction and how these factors relate to the organizational effectiveness of hospitals.

## Literature

### Complexity of healthcare management

Organizational management in the healthcare sector is different from and therefore difficult to compare with that of industrial organizations [11]. Its distinguishing factors include the following [12]:

- high variability and multidirectionality of work, which make it more difficult to regulate and measure performance and quality;
- most activities should be performed immediately and precisely, with minimum scope for error;
- individual work activities are highly independent and require perfect coordination between various groups of professionals;
- the education of medical staff is highly specialized, and they feel more loyal toward their professional group rather than toward their organization;
- medical doctors make the greatest contribution to overall healthcare services, and they are therefore committed to autonomy and only reluctantly submit to effective organizational and executive supervision; and
- there are two types of professional subordination in hospitals: clinical and administrative.

A different mindset (mentality) of executives and medical staff, further amplified by the diverse nature of their work, is another source of conflicts in the management of healthcare professionals. The work of a medical doctor is based on science and rationality, whereas management is inherently less deterministic and more open to free interpretation. Hospital doctors are empowered to decide how to provide healthcare services as well as to choose whether and which resources to use. The executive director of a hospital may find it difficult to regulate, measure, and control the work of medical professionals who are free to make autonomous decisions [13]. The differences are evident in the preference of hospital performance indicators. The executives prefer structural indicators related to organization and its output, such as formal qualification and number of staff, which they can influence, while physicians opt for process-based indicators related to outcomes that they can control, such as proper diagnosis [2].

Many authors also argue that mismanagement or poor governance is the main obstacle to improve healthcare performance [6, 14]. With the absence of a clearly defined strategy of HR management, many countries are facing employment instability that threatens to paralyze the healthcare system [15]. A severe shortage of medical professionals, especially medical and nursing staff, has become a global problem [16]. A downward trend in physician employment figures is also seen in Poland [17]. The current number of physicians per 1000 population is only 2.3 and is the lowest in the European Union, with the average doctor-population ratio of 3.5.

Effective work motivation of the medical staff may be particularly relevant for improving the overall healthcare performance; here, the role and skills of hospital management can hardly be overestimated [18]. The rule is simple: if the executives endeavor to satisfy the essential workplace-related needs of medical staff, healthcare professionals will care more about reputation of their institution and will be more likely to recognize and satisfy patient needs [19].

### Work motivation among medical doctors

The significance of work motivation is more evident among medical staff than among other public service employees. An aspect common for all medical doctors is that they work with patients who require special care and attention. This implies commitment and dedication as well as the ability to cope with the mental burden of having to deal with difficult patient experiences [20]. WHO suggests that the motivation of healthcare professionals should be considered as the main indicator of the quality of healthcare services [21]. Physicians who are more engaged in their work obtain better treatment outcomes as well as higher personal and patient satisfaction than those who are less motivated [5, 22].

Given the scarcity of literature on this subject, there is less information on the factors that influence the commitment of medical staff as opposed to nursing staff [23]. An important study found that achievements (meaning of work, respect, and interpersonal relations) constitute the main motivation factor for medical doctors, followed by remuneration, cooperation, and work attributes [24].

It should be highlighted here that individuals who choose to become medical doctors are very focused on their professional success and are more interested in motivation drivers. For example, they want to know how well they perform [25, 26].

### Performance feedback

To gain competitive advantage, hospitals should conduct more training and development programs for medical staff [27, 28]. Performance feedback is particularly important for medical doctors. Knowledge and skills underpinned by clinical experience are the fundamental drivers of this profession [29]. Performance feedback is the starting point in planning professional development. It also helps to use reasonably the capacity of medical staff and contributes to better overall organizational performance [29].

As a rule, hospitals in Poland do not have any formal systems for setting goals, criteria, and ratings in HR management [29]. They most often operate according to informal principles, which can hardly be considered as a sound basis for decision-making related to HR [30].

It is important to create formal assessment processes that are beneficial for personal development and recognize individual accomplishments of employees. In the UK, National Health Service uses Individual Performance Review (IPR) to evaluate the performance of medical staff. This method helps to meet professional requirements in terms of goal-setting or employee development review, even among high achievers. David Wigley argues that the IPR system should cover both individual motivation [31] and external motivation [32], and then combine it with organizational development programs to create an appropriate organizational culture [31]. IPR is a tool that enables achievement of high productivity, improved performance, and overall commitment [32] as well as improved behavior and professional autonomy of medical doctors and their involvement in the ongoing changes [33].

HR management systems that understand and value feedback – the process of providing information on performance at work and involves performance review and goal-setting – have a positive effect on the behavior of medical staff and clinical care indicators [33–35].

NHS also reviews clinical outcomes at the individual level through “rigid systems” by measuring and publicly disclosing data on the obtained treatment outcomes [36–39]. Clinicians are awarded bonuses for their positive performance [32].

Researchers have highlighted the significance of evaluating performance (unless something can be measured, it cannot be improved) [39]. They have also indicated the lack of systems to monitor and improve the performance of medical doctors [40]. Hospitals need better performance metrics and more widespread implementation of research and remedial action plans.

### Motivation of medical staff versus organizational performance of hospitals

According to the results of studies exploring the link between the motivation of medical doctors and the organizational performance of hospitals, the commitment and support of medical staff are the critical determinants of hospital performance whenever changes are introduced for improving the hospital’s organizational performance, value, or quality [7–9].

To boost the motivation of medical staff and hence to safeguard proper performance at both individual and hospital levels, healthcare centers should implement organizational and management processes to align the needs of healthcare professionals with organizational goals [41].

## Methods

### Main objective

This study examined the influence of work motivation of medical doctors on the organizational performance of public hospitals.

**Fig. 1:**
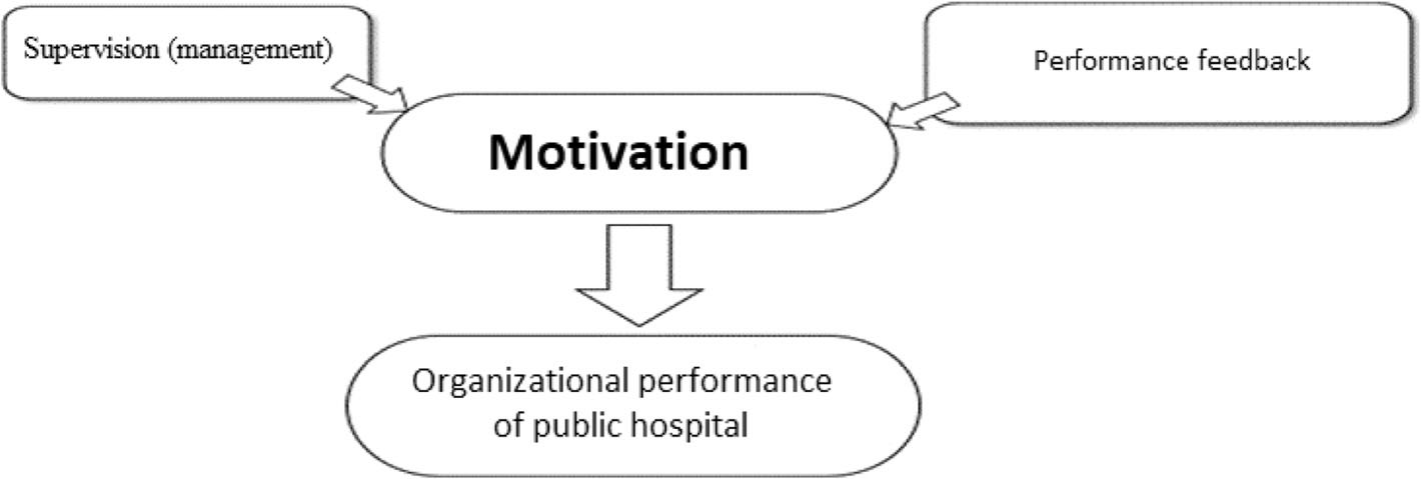
Conceptual framework of the study

The concept of the study is presented below (Fig. 1).

Motivation is an independent variable and was examined using two factors: quality of supervision and performance feedback. Organizational performance of public hospitals is the dependent variable.

The following hypotheses were investigated on the basis of relevant literature and the proposed model of the study:

- H1: There is a relationship between the quality of supervision of medical doctors and their work motivation,
- H2: There is a relationship between providing feedback about the performance of medical doctors and their work motivation, and
- H3: There is a relationship between work motivation of medical doctors and the organizational performance of hospitals.

### Data sampling and research tools

First, 22 public hospital departments/clinics in Warsaw, Poland, were randomly selected. Only general (multispecialty) public hospitals were included in the survey. Next, the survey was conducted among all physicians who agreed to participate in the study (*n* = 249) and worked in the listed departments. Among the approached physicians, 5% (*n* = 13) refused to participate in the survey.

WHO questionnaires were used, either as an auditorium employee survey distributed among physicians on duty, during a briefing or scientific consultation, accompanied by a short discussion of this research project or as a direct survey administered to physicians at their place of work, e.g., in a surgical room or a break room.

These measures were collected from a selected occupational group – medical doctors – in order to gather information about the perception of motivation factors and organizational effectiveness variables by the stakeholders of the same hospital. Noninclusion of other occupational groups in the analysis was a limitation attributed to the study method.

The factors that influenced the attitude to work (attitude to work implies satisfaction and dissatisfaction) were identified according to Herzberg’s motivation theory, whereas the variables of organizational performance were examined according to the McKinsey framework.

### Herzberg’s and McKinsey’s concepts

F. Herzberg model, or the two-factor theory, is the most universally used motivation theory in management [24, 42–47] and the most common methodology applied by organizations [42]. Herzberg uses a two-dimensional approach to determine motivation at the workplace: (1) motivating factors that lead to job satisfaction and (2) a separate set of demotivating factors that cause job dissatisfaction [48]. Herzberg also identifies hygiene factors that do not in themselves motivate employees; however, if they are missing at the workplace, they would cause dissatisfaction with work. Hygiene factors include company policy and administration, supervision, relationships with superiors, working conditions, salary, relationships with same-rank co-workers, personal life, relationships with subordinates, status, and security [49]. These elements can be tailored to minimize work dissatisfaction. The motivating factors (motivators/satisfiers) involve achievement, recognition for achievement, the nature of work itself, responsibility, career advancement, and opportunities for growth. The motivation-hygiene theory suggests that “job enrichment” is necessary to improve employee performance [49] and that it should not be a one-off exercise, but rather a continuous process coordinated by the management. Herzberg suggests that performance feedback be provided to secure and create conditions for professional growth, to empower employees to self-organize their work, and to discuss the goals achieved by the employees. Herzberg argues that even a small amount of time and money invested in job enrichment will translate into employee satisfaction and economic effects that will ultimately benefit the entire society. The skills of employees should also be effectively utilized [49].

In this research project, the relationship between motivation and organizational performance is based on McKinsey’s 7S framework or the management model for organizational effectiveness. This model postulates that organizational effectiveness depends on several factors [50]. The model specifies seven factors (7S) as the main variables that shape organizational effectiveness [50]: shared values, strategy, structure, system, staff, style, and skills. Thus, organizational effectiveness in McKinsey’s model is the net effect of interactions among these variables.

### Statistical analysis

The examined factors of job motivation and organizational effectiveness were grouped into stens with an assigned value calculated as an arithmetical mean of individual components of a sten. Normal distribution of the stens was verified with the Kolmogorov–Smirnov test. Next, 95% confidence intervals of stens were calculated and used for comparison.

Significant differences among the examined stens according to physician’s specialty were analyzed with Student’s t-test for independent samples. Spearman’s rank correlation coefficient was used to analyze the relationship between various aspects of work and the mean organizational effectiveness. The significance level was set at < 0.05. Statistical analysis was performed with SPSS Statistics 19.0 software (IBM).

### Ethical considerations

This study was approved by the Ethics Committee of the Medical University of Warsaw (Approval No. AkBE/116/15). The researchers duly informed heads of hospital departments and medical doctors about the study. The contact details of the researchers and research information were included in the questionnaires. Participation in the study was voluntary, and the questionnaires were completed anonymously.

## Results

**Fig. 2:**
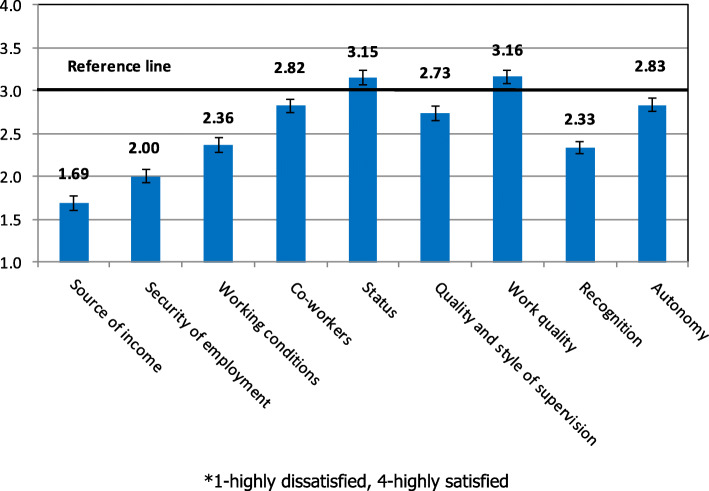
Assessment of factors affecting work attitudes

The factors that influenced attitude to work were evaluated first. Questions on various aspects of work according to Herzberg’s motivation theory were divided into nine groups – stens (Fig. 2).

Quality of Work and Status were found to be the greatest source of job satisfaction (a score of 3 on a 4-point scale). The scores of Autonomy, Co-workers, and the Quality and Style of Supervision were also high and above the reference line (2.5). The respondents were least satisfied with the Source of Income (1.69) and Stability of Employment (2.00).

**Fig. 3:**
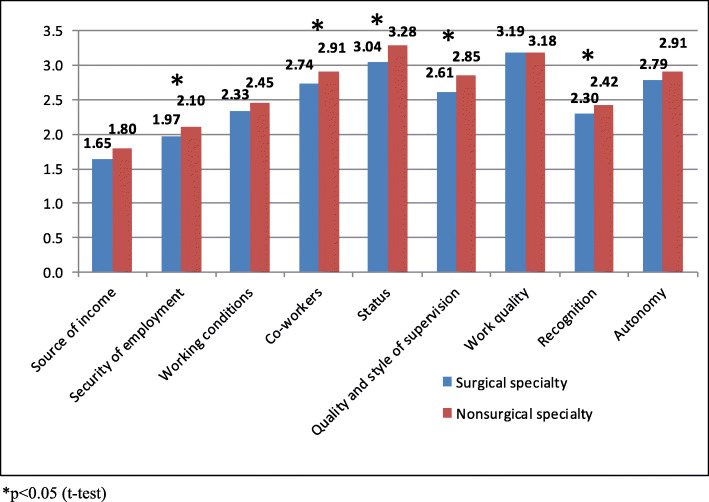
Assessment of factors affecting work attitudes according to physicians’ specialty

The study showed significant differences in ranking individual aspects of work by physicians from two different specialties (Fig. 3).

All the analyzed stens (apart from Quality of Work) were ranked lower (but not all of them were significant) by surgeons. Significant differences were noted for the following stens: Security of Employment (*p* = 0.027), Co-workers (*p* = 0.009), Quality and Style of Supervision (*p* = 0.002), Status (p = 0.009), and Recognition (*p* = 0.014). The most notable differences (0.24) were identified for Status and Quality and Style of Supervision, while the least difference (0.12), which was significant, was observed for Recognition.

**Fig. 4:**
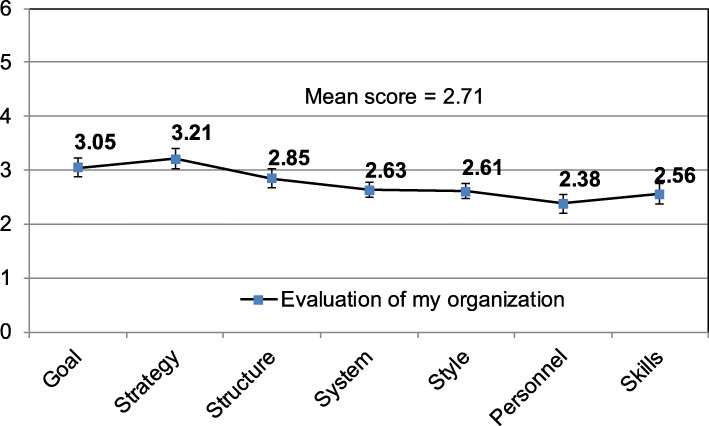
Assessment of organizational performance variables according to the criteria of McKinsey framework

Next, the organizational performance of public hospitals (Fig. 4) was analyzed. In general, the respondents gave low ratings to each variable of organizational performance of their hospitals.

Strategy and Goal were scored the highest at 3.21 and 3.05, respectively, while employees were ranked the lowest (2.38). It is, however, worth noting that all scores were relatively poor or average. The neutral level was 3.5.

It should also be noted that the ratings of surgeons for all 7 factors were significantly lower than those of nonsurgeons (Table 1).

**Table 1:** Evaluation of organization performance variables by physicians according to their specialty (surgeon vs. nonsurgeon) on a scale of 1 to 6

Organization characteristics	Surgical specialty				Significance
	No		Yes		
	Mean	Standard error	Mean	Standard error	
Goal/Shared values	3.27	0.11	2.76	0.13	0.003
Strategy	3.47	0.12	2.87	0.14	0.001
Structure	3.06	0.12	2.56	0.12	0.003
Systems	2.92	0.10	2.26	0.09	< 0.001
Style	2.94	0.11	2.18	0.09	< 0.001
Staff	2.70	0.13	1.95	0.11	< 0.001
Skills	2.91	0.13	2.09	0.12	< 0.001
Total	3.00	0.10	2.32	0.09	< 0.001

**Fig. 5:**
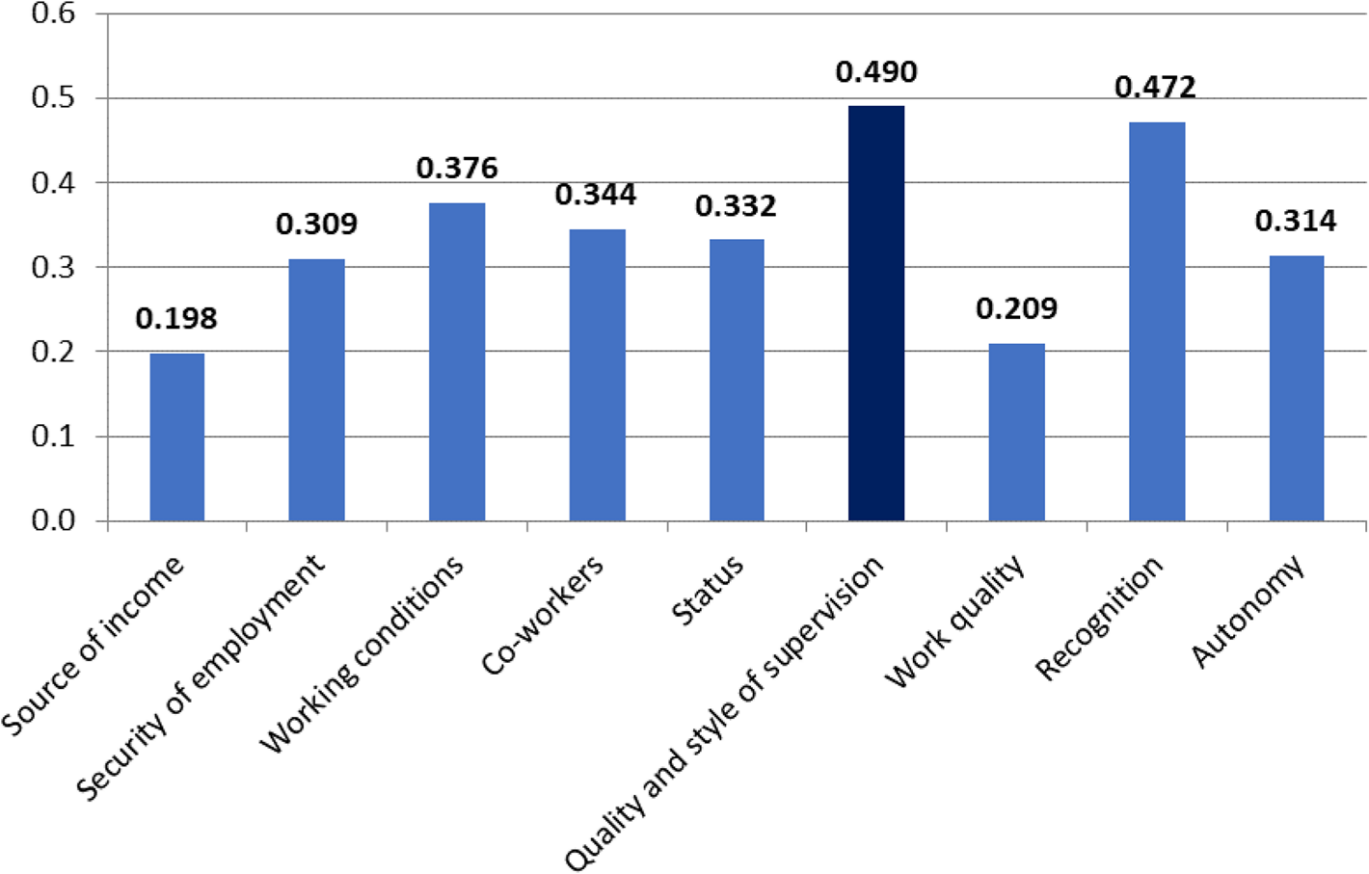
Coefficients of Spearman’s rank correlation between factors affecting work attitudes and the mean assessment of organizational performance

Next, we examined whether a statistically significant correlation existed between the work motivation factors of medical doctors and the organizational performance of public hospitals in which they worked (Fig. 5).

All the examined aspects of work were significantly correlated with the hospital’s organizational performance. Quality and Style of Supervision and Recognition were most strongly correlated with organizational performance.

**Fig. 6:**
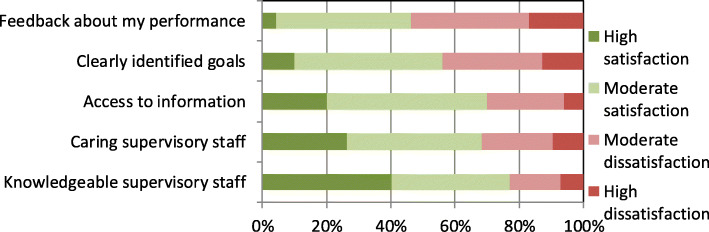
Assessment of a group of factors affecting work attitudes attributed to Quality and Style of Supervision

Further analysis revealed how the respondents evaluated individual aspects of Quality and Style of Supervision (Fig. 6).

Regarding Quality and Style of Supervision, the respondents were least satisfied with Performance Feedback and Assignment of Clear Goals. This implies that if physicians felt more satisfied with these aspect(s) of their work, they would more positively assess their organization.

## Discussion

The evaluation of factors that medical doctors working in public healthcare consider to be the determinants of job satisfaction can provide valuable insights on whether any measures are necessary to improve the conditions that cause their dissatisfaction with work and consequently improve healthcare services in general [51, 52]. It is known that neither job satisfaction nor motivation can be easily captured; however, once they are measured, they can help to maintain and improve the performance of healthcare professionals [53, 54]. Experts in HR management increasingly recognize motivation as the key feature in predicting the behaviors or aspirations of individual employees.

The results of the present study indicate that among ***various aspects of work***, the main determinants of job satisfaction among medical doctors include Quality of Work (the essence of work), Status (respect from co-workers and respectable social status), and Autonomy. Source of Income, Security of Employment, and Recognition were deemed to be the greatest source of dissatisfaction with work. A survey conducted among 67 physicians working at a public hospital in Cyprus [24] in 2010 showed similar results, although the examined categories were placed in a slightly broader context; the meaning of work, respect, and interpersonal relationships were ranked the highest in the “Achievements” category. The “Co-workers” category was ranked second and featured five items: teamwork, sense of pride, recognition, superiors, and integrity, followed by “remuneration” and “training”. Comparable results were reported by N. Kontodimopoulos et al. [55] in 2009, in which achievements (categorized as in the study by P. Lambrou [24]) were found to be the most important motivation factor among 354 surveyed physicians. The question of autonomy was viewed differently – it was ranked third in the discussed study and was considered to be among the least important motivation factors in the study performed in Greece. Kisa et al. [56] reported similar results in 2009 [56]. In a study of 351 physicians working in public hospitals in Turkey, the nature of work (diversity of cases, working with people, social importance of the work performed, and patient care) was considered most satisfying. The least satisfying aspects included the lack of career prospects, knowledge development, and involvement in organizational and administrative decision-making. The respondents were also very dissatisfied with remuneration. Vasconcelos et al. studied a group of 141 physicians working in a public hospital and found that a good relationship with other medical professionals was the greatest source of job satisfaction. Apart from this aspect, the respondents also appreciated the opportunities for career development and research, social prestige attributed to working for a prominent institution, and the hospital’s affiliations with a medical university [57]. Linzer et al. [58] also reported the beneficial effects of positive relationships among medical staff and job satisfaction. Other motivating factors, i.e., providing help to patients or personal and professional values, were also emphasized in qualitative interviews conducted in Benin and Kenya; the respondents from Kenya also highlighted the importance of recognition [59]. Studies performed in Vietnam [60] yielded different results in terms of ranking of individual motivating factors. Here, the relationships with superiors and co-workers were given equally high importance, but the ranking of remaining items varied considerably. Security of employment and salary were ranked third at a level equivalent to “autonomy” in the present study. Regarding demotivators, healthcare professionals clearly agreed that low wages, lack of information, and absence of training were causes of dissatisfaction. Employment instability (security of employment) is a missing dissatisfaction factor when compared with the present study. A research project in Tanzania [61] also indicates the importance of incentives for work among healthcare professionals, particularly the importance given to appreciation by superiors, co-workers, and the community as well as stability of employment, salary, and training. Similar levels of dissatisfaction with work were observed in a group of 132 physicians in Pakistan. Apart from remuneration, these physicians mainly complained of stress at work, poor opportunities for enhancing their medical knowledge, and the lack of individual career development paths [62]. Rosta in 2006 [63] reported that 1917 hospital physicians from Germany expressed only moderate satisfaction with their work. Working hours (3.25), Remuneration (3.59), Physical Conditions of Work (3.96), and Recognition for Good Performance (4.08) were among the highest ranked factors affecting work performance. The study concluded that German physicians are less satisfied with work than their counterparts in England, New Zealand, and Norway. Nylenna surveyed 1174 physicians in Norway in 2005 [64] and confirmed that the job satisfaction levels increased over the last decade. The researchers even claimed that Norway challenges the worldwide trend of high dissatisfaction levels among medical staff. American researchers Hinami et al. [65] also demonstrated high levels of job satisfaction among 816 hospital physicians. They found that 62.6% of the respondents were highly satisfied with work, with score > 4 on a 5-point scale. The physicians were most satisfied with the quality of services provided and the relationships with staff and other healthcare professionals, and they were relatively satisfied with the organizational culture, autonomy, remuneration, and leisure time.

***Medical specialty*** is one of the key determinants of the nature and conditions of medical practice and job satisfaction [64, 66]. The present study revealed differences in the evaluation of work-related aspects (and variables of organizational performance) among surgeons and nonsurgeons. Surgeons who were significantly less satisfied with their work showed a higher number of statistically significant correlations than nonsurgeons. Similar results were reported by J.P. Leigh et al. [67] in a group of 6590 physicians working in the USA. Six out of 10 medical specialties that showed higher job satisfaction were nonsurgical specialties, while 5 out of 11 specialties with the lowest levels of job satisfaction were surgical ones. In the study of Rosta [63], radiologists were most satisfied and surgeons were least satisfied with their work. Job satisfaction levels among urologists and internists were below average. In other reviewed studies, there was no consensus regarding the key factors underlying job dissatisfaction, which resulted in a high turnover of medical staff in correlation with medical specialties [24, 68–72]. Likewise, P.O. Vasconcelos et al. found no correlation between job satisfaction and medical specialty [57]. However, H. Cerwenka et al. observed a correlation between job satisfaction and specialty in a study on 667 physicians with surgical specialties working in Austria [73]; here, only 37% of physicians with surgical specialty were satisfied with the conditions of their work. Excessive bureaucracy, remuneration, and long working hours were considered the most dissatisfying aspects of work.

It is particularly important to identify the key ***motivating factors that influence the relationship between job satisfaction and organizational performance*** and the main correlations among them [74]. Evidence from behavioral and social research suggests that job satisfaction and performance at work are positively correlated [75]. According to Mascia, job satisfaction among medical doctors is highly dependent on the organizational culture [76]. C.C. Demir et al. conducted surveys among 635 physicians from one of the largest military hospitals in Turkey, and they claimed that work-related factors have a greater influence on the physicians’ commitment to their current organization (i.e., loyalty) than their personal qualities, which consequently determines the fulfillment of organizational goals [23].

The relevant literature also describes determinants of job satisfaction and their influence on the overall performance of organizations [74, 77]. ***Management and supervision*** were found to be the predictors of job satisfaction and organizational commitment of hospital staff. A meta-analysis by R. Hogan et al. revealed that the head (leader) of an organization played a critical role in shaping job satisfaction and organizational performance, and therefore, organizations consider leadership very seriously, as it can contribute to better team cooperation and patient care [78]. Other authors suggest an equally strong relationship between leadership and the quality of healthcare [79, 80]. H.M. Elarabi [81] also demonstrated a positive correlation of all work-related factors and the general performance of a hospital in Tripoli. The surveyed physicians also highlighted the importance of treatment at work, convenience of work, and incentives and remuneration. The present study clearly demonstrates a strong and positive correlation between satisfaction with various aspects of work and the assessment of variables of organizational performance. Quality and Style of Supervision was most strongly correlated with organizational performance, which is reflected in the discussed literature data.

The quality of supervision and ***performance feedback*** from the management are the motivating factors with a lasting effect on attitude to work. Not only do they enhance job satisfaction, but they also improve organizational and staff performance [25]. In the present study, 54% of the study population were relatively dissatisfied with their performance feedback. Medical doctors with a nonsurgical specialty formed a significant part of respondents who were relatively satisfied. Similar conclusions were drawn from a survey of surgical interns in Wales, 70% of whom were not subjected to regular review [82]. In a study of surgical residents in Germany, only 18% of the respondents believed that the hospital was interested in their progress [83]. The studies also indicate another important issue, which is mainly related to doctors in surgical training. It is alarming that despite the legal requirements, many junior physicians are not provided regular feedback either from the head of department or from the supervising physician. This appears to be a worldwide problem [73]. Likewise, a study of military doctors conducted by S. Chaudhury in 2002 revealed that HR policy was the key determinant of job dissatisfaction, including employee performance reviews and poor opportunities for promotion as well as suboptimal use of the capacity of medical staff [51].

Although performance can be influenced by different types of motivation factors, motivation is undeniably one of the preconditions for performing a good job [1].

## Strengths and limitation of the study

The random selection and relatively robust sample size are clear strengths of this study, together with the use of the WHO questionnaire and the two relevant theories that frame the study and help to explain the results. Comparison of the results of the present study and the results of research conducted in highly developed and less developed countries confirms the international range of complex problems faced by contemporary healthcare systems. The results of this study also confirm the need to conduct further similar studies and to expand the scope of work to include hospitals in smaller towns. Limitations of this research include the use of bivariate tests of significance rather than multivariate models that allow for the inclusion of relevant control variables.

## Conclusions

Motivation is the driving force of success in any organization. This is especially true in the healthcare environment in which the performance of individual healthcare units largely depends on the commitment and dedication of healthcare staff [23, 84]. However, motivation in itself is not sufficient to provide high work efficiency. It must be accompanied by high standards of management which can ensure that the efforts of the staff are used as effectively as possible. The results of analyses confirm that the quality of supervision and work motivation of medical staff are interrelated. It is also worth highlighting that surgeons are significantly more likely to be more dissatisfied with most aspects of work and organization as compared to nonsurgeons. Hence, programs designed to improve motivation of surgeons should be modified accordingly.

It should also be acknowledged that to enhance the organizational performance of hospitals, the measures to drive motivation may have to be aligned with other aspects of management. An analysis of correlations revealed a strong relationship between motivation driven by organizational aspects and the mean rating of organizational performance, with Quality and Style of Supervision being most strongly correlated. Therefore, hospital performance may improve if physicians are more satisfied with their superiors, rewards, trainings, and career opportunities.

The results of further analyses confirmed the existence of a relationship between Performance Feedback and work motivation of medical doctors. A majority of the physicians surveyed (more than 50%) mentioned that they were least satisfied with Performance Feedback as an aspect of Quality and Style of Supervision. It is thus necessary to introduce formal assessments of physicians’ performance, which could also be used as a measure to recognize individual achievements. Therefore, consideration should be given to whether IPR tools should be introduced to hospitals, as was done in the NHS, to facilitate goal-setting and performance rating and to support staff development. It is also advisable to rapidly share more comprehensive information about achievements of individual physicians at departmental and hospital levels.

## Data Availability

All data can be requested from the corresponding author.

## Abbreviations

HR: Human Resources
IPR: Individual Performance Review
NHS: National Health Service
UK: United Kingdom
USA: United States of America
WHO: World Health Organization
